# Groundwater extraction reduces tree vitality, growth and xylem hydraulic capacity in *Quercus robur* during and after drought events

**DOI:** 10.1038/s41598-021-84322-6

**Published:** 2021-03-04

**Authors:** Georgios Skiadaresis, Julia Schwarz, Kerstin Stahl, Jürgen Bauhus

**Affiliations:** 1 Chair of Silviculture, Institute of Forest Sciences, University of Freiburg, Tennenbacherstrasse 4, 79085 Freiburg im Breisgau, Germany; 2 Chair of Environmental Hydrological Systems, University of Freiburg, Friedrichstrasse 39, 79098 Freiburg im Breisgau, Germany

**Keywords:** Forest ecology, Forestry, Environmental sciences, Forest ecology, Forestry

## Abstract

Climate change is expected to pose major direct and indirect threats to groundwater-dependent forest ecosystems. Forests that concurrently experience increased rates of water extraction may face unprecedented exposure to droughts. Here, we examined differences in stem growth and xylem hydraulic architecture of 216 oak trees from sites with contrasting groundwater availability, including sites where groundwater extraction has led to reduced water availability for trees over several decades. We expected reduced growth and xylem hydraulic capacity for trees at groundwater extraction sites both under normal and unfavourable growing conditions. Compared to sites without extraction, trees at sites with groundwater extraction showed reduced growth and hydraulic conductivity both during periods of moderate and extremely low soil water availability. Trees of low vigour, which were more frequent at sites with groundwater extraction, were not able to recover growth and hydraulic capacity following drought, pointing to prolonged drought effects. Long-term water deficit resulting in reduced CO_2_ assimilation and hydraulic capacity after drought are very likely responsible for observed reductions in tree vitality at extraction sites. Our results demonstrate that groundwater access maintains tree function and resilience to drought and is therefore important for tree health in the context of climate change.

## Introduction

Climate change poses profound threats to forest ecosystems globally. A plethora of studies have reported widespread forest decline in most forest types of the planet, which has been attributed to increases in frequency and intensity of droughts and associated disturbances^1–4^. Indeed, drought affects all types of forests including in regions where tree growth is generally not considered water limited such as groundwater-fed forests^1,2,4^. Yet, there have been only very few studies on drought effects in these wet forest ecosystems. The majority of research in this field has been conducted in drier areas or even towards the limits of species distribution areas, which are often deemed more vulnerable^3,5,6^. However, results of the few existing studies indicate that despite their overall adequate water availability, trees in wet forests are indeed also vulnerable to periods of extreme water deficits^1,5–8^.

Floodplain and groundwater-fed forests that depend on groundwater to cover their water demands are typically characterized by high productivity, partly due to ample water availability^9^. These forests are also extremely valuable for their biodiversity and unique ecosystem services and functions such as buffering of floods, stabilization of river banks and water quality improvement^10–15^. Land-use change, groundwater extraction and river regulation have already resulted in reduced groundwater availability for adjacent ecosystems in many parts of the globe^14^. In addition, climate change has been suggested to further reduce the quantity of groundwater resources and their availability for dependent ecosystems^16,17^. The magnitude of direct climate change effects is difficult to predict. However, groundwater extraction rates for agricultural, industrial and domestic uses will most certainly increase during more frequent and intense drought events in future^17–19^. This combination of direct and indirect effects of climate change will expose these forest ecosystems to potentially dramatic decreases in water availability^15^.

The consequences will largely depend on the resilience of trees in response to long-term (e.g. decrease in groundwater levels) and short-term water deficits during drought events. Previous studies at sites with reduced groundwater access reported widespread dieback of floodplain *Eucalyptus* forests in southeastern Australia^9^, delayed or incomplete recovery in the years following drought for 17 common tree species in mesic temperate forests in midwestern and eastern US^6^ and even reduced ability to take advantage of favourable growing conditions in *Q. robur* dominated former floodplain forests^20^. However, we lack an understanding of the mechanisms that underlie this impaired growth performance particularly at the species level. This is essential to explain observed declines in tree vitality and identify populations most vulnerable to climate change^21–24^.

The study of wood anatomical properties and plant hydraulics is central to understanding how species respond to environmental changes^25–27^. Information encoded in the anatomy of tree-rings is the result of several interacting factors related to wood formation, including genotype, hormones and environmental conditions^27,28^. Environmental conditions such as droughts during wood formation directly influence the characteristics of xylem cells through the availability and translocation of photoassimilates^27–29^. At the same time, the structure and function of the water transport systems within trees place a physical limit to plant functions such as photosynthesis and transpiration that cannot be exceeded^30^. This strong connection between xylem anatomy, plant function, and water availability makes the study of xylem hydraulic architecture highly suitable to assess which populations could be most vulnerable to drought^26,30,31^.

For ring-porous species such as the most wide-spread European oak species, pedunculate oak (*Quercus robur* L.) and sessile oak (*Quercus petraea* (Matt.) Liebl.), xylem water transport relies strongly upon earlywood vessels formed in the same year^27,28^. This reliance on newly formed vessels is very likely associated with winter embolism of previous years’ large conduits, which means that these species need to rebuild their disrupted hydraulic pathway each spring before leaf flush^32,33^. In addition, embolism and tylosis formation due to spring or early summer drought might disrupt the newly formed earlywood vessels, in which case water transport in ring-porous tree species may rely solely on latewood vessels^34^. Thus, a high plasticity in response to prevailing climatic conditions can be expected for these species^26^. Several studies report distinct environmental signals present in annual series of ring-width and anatomical features (mostly related to size and arrangement of earlywood vessels) of pedunculate oak and other oak species^35–41^. The climate signal reflected in xylem anatomical variables is often associated to climatic conditions prevailing in a shorter time-period than that of ring-width variables^27,39,42^. Overall, studies on ring-porous oaks report strong associations between earlywood vessel characteristics and climatic conditions in the previous growing season or early spring when earlywood vessels are formed^39,42^.

Several studies have examined environmental signals encoded in series of earlywood vessels in ring-porous oak species and demonstrated their great physiological relevance. However, to our knowledge, these studies have not examined short-term responses of wood anatomical properties to drought and how these are affected by reduction in groundwater availability. We hypothesized that:

- Tree-ring and vessel related anatomical variables of oak trees are strongly related to soil–water availability at the time of their formation;
- Groundwater extraction reduces resilience of radial growth and hydraulic capacity of oaks to drought;
- Oak vitality is directly related to the ability of trees to restore hydraulic capacity following drought.

## Results

### General results

Although we selected an equal number of declining and healthy trees per stand for wood anatomical measurements, the distribution of trees among vigour classes differed across sites with varying groundwater availability (Table 1). Almost half of the initially sampled trees at extraction sites were classified as declining (class 2 or 3). In contrast, only 30% and 26% of trees were in declining condition at no extraction and upland sites, respectively. Only 17% of trees at extraction sites were vital (class 0) while approximately 10% more vital trees were found at no extraction and upland sites (Table 1).

**Table 1:** Percentages and total number of sampled trees per vigour class at each site.

Site	Healthy		Declining		Total number of trees per site type
	Vigour class 0	Vigour class 1	Vigour class 2	Vigour class 3	
Extraction	**17.1**%	**31.7**%	**27.5**%	**23.7**%	240
No extraction	**27.9**%	**42.1**%	**20.7**%	**9.3**%	140
Upland	**28.8**%	**44.3**%	**22.8**%	**4.0**%	149
Totals	123	201	129	76	529

There were no statistically significant differences in tree diameter and height among the different vigour groups and sites, except for healthy trees at upland sites which were significantly taller than declining trees at the extraction sites (Supplementary Table S1).

### Tree-ring and wood anatomical features

The descriptive statistics calculated for both ring-width and vessel variables indicated a high common signal present in the developed superregional (i.e. averaged across the three investigated regions) chronologies (Supplementary Table S2).

We observed strong correlations among the nine ring-width and vessel related variables (see Supplementary Fig. S5). Strong positive correlations were observed among the three ring-width chronologies: Ring width (RW) was strongly correlated with both earlywood (EW) and latewood (LW) (*r* = 0.91 and *r* = 0.87, respectively), while the correlation between EW and LW was weaker (*r* = 0.62). All three ring-width variables showed strong negative correlations with total vessel area expressed as percentage of total ring area (TVA%). LW showed a strong negative correlation with TVA%. Vessel density (VD) was negatively correlated with LW (*r* = − 0.91). The two proxies of water transport efficiency (Dh and Ks) were strongly correlated with each other and both showed strong positive correlations with EW and TVA.

### Hydro-climatic sensitivity of tree-ring and vessel related variables

#### Hydro-climatic sensitivity of ring-width chronologies

**Figure 1:**
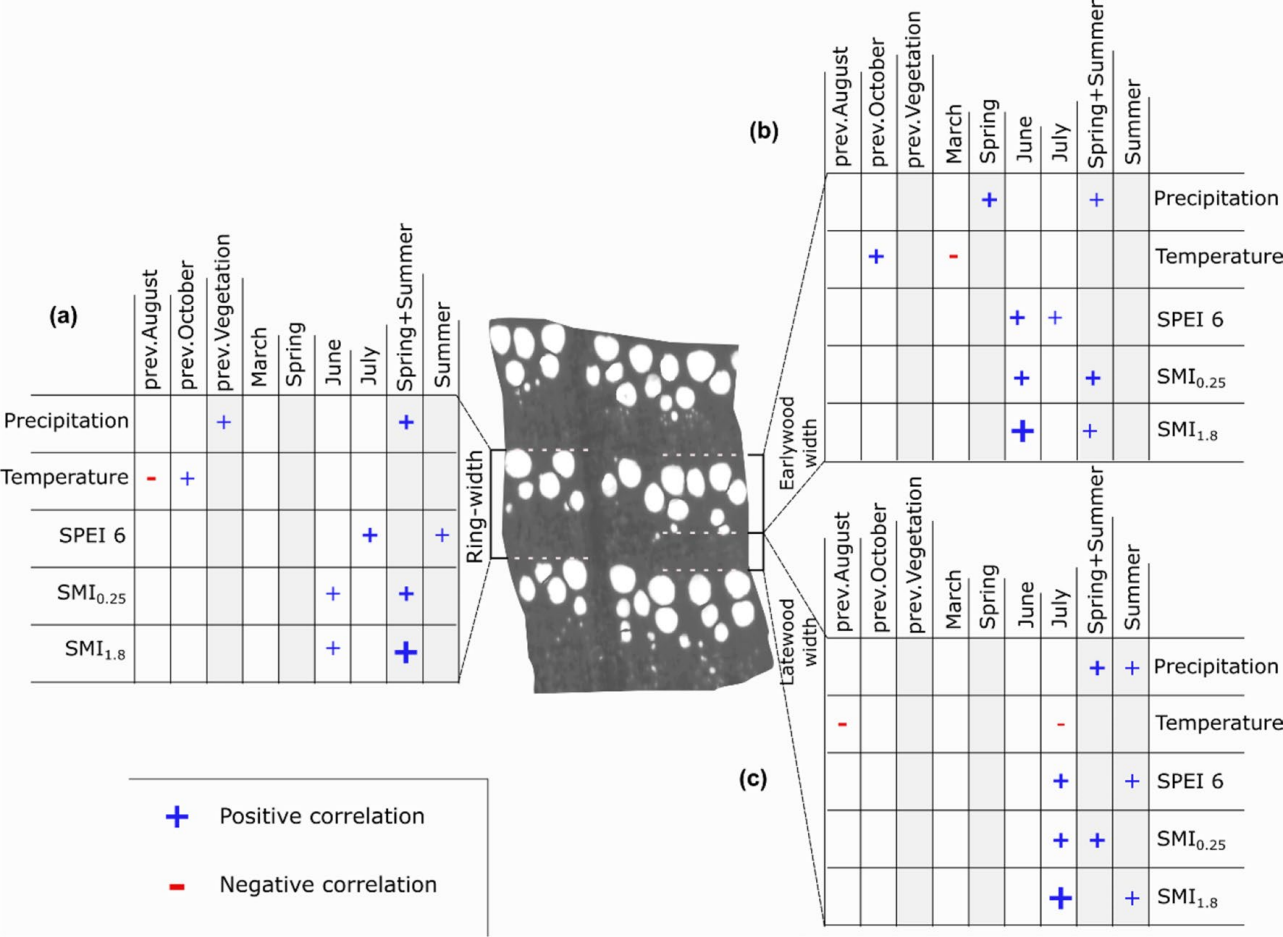
Strongest correlations between hydro-climatic variables (precipitation, temperature, standardized precipitation evapotranspiration index (SPEI 6), upper and total soil moisture index (SMI)) and ring-width chronologies ((**a**): ring-; (**b**): earlywood; and (**c**); latewood-width). Columns highlighted in grey show seasonal averages. SMI: Soil moisture index. Symbol size indicates correlation strength for each ring-width variable.  The strongest correlation in absolute terms was observed betwen earlywood and June SMI_1.8_ (*r* = 0.6), while the lowest correlation between latewood and July temperature (*r* = − 0.25).

The strongest correlations between ring-width chronologies and hydro-climatic variables were observed for the soil moisture anomaly index of the total soil column (SMI_1.8_) (Fig. 1). RW was strongly positively correlated (*r* = 0.58) with combined spring and summer SMI_1.8_ (Fig. 1a and Supplementary Fig. S6). Average precipitation and combined spring and summer SMI_0.25_ seemed to play an important role for RW as well. EW was strongly associated with climatic conditions prevailing in spring and June (Fig. 1b). SMI_1.8_ in June and average of SMI_1.8_ of spring and summer months combined, showed the highest correlations with EW (*r* = 0.6 and *r* = 0.59 respectively). SMI_0.25_ of the same month and period was strongly correlated with EW, although with slightly lower correlation coefficients than SMI_1.8_ (Supplementary Fig. S6). Average precipitation of spring months as well as of spring and summer months combined were also positively correlated with EW. However, these correlations were weaker than with SMI_1.8_. Correlations between LW and hydro-climatic variables were weaker than with RW and EW. SMI_1.8_ in July and the average of summer months showed the highest correlations with LW (*r* = 0.47 and *r* = 0.45, respectively). Interestingly, climatic conditions in the year prior to ring formation played an important role for ring-width variables. Average precipitation in the vegetation period prior to ring formation was positively correlated with RW. Temperature in previous October correlated positively with RW and EW. In contrast, August temperature in the previous year was negatively correlated with RW and LW.

#### Hydro-climatic sensitivity of vessel chronologies

**Figure 2:**
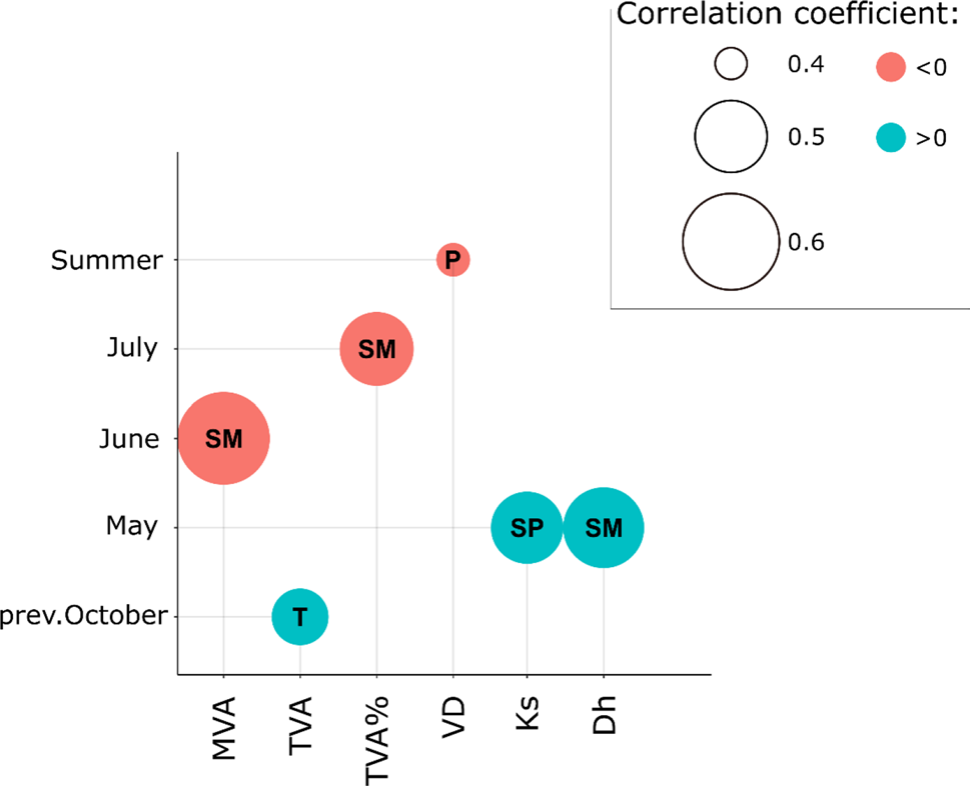
Strongest relationships (Pearson’s correlation coefficient) between hydro-climatic variables: precipitation (P), temperature (T), standardized precipitation evapotranspiration index (SP), SMI_1.8_ (SM)) and vessel related variables: mean vessel area (MVA), total vessel area (TVA), total vessel area expressed as percentage of ring area (TVA%), vessel density (VD), hydraulic diameter (Dh) and the theoretical hydraulic conductivity (Ks).

Mean chronologies of vessel related variables showed strong associations to climate variables and soil moisture anomalies (Fig. 2, Supplementary Figs. S7 and S8). MVA was significantly negatively correlated with SMI_1.8_ in June. TVA showed the strongest correlations with temperature in October of the previous year (*r* = 0.43) and with SMI_1.8_ in May (*r* = 0.40). TVA% was strongly negatively associated with July SMI_1.8_. Likewise, VD showed a significant negative correlation with average precipitation during summer months. Both variables related to theoretical hydraulic conductivity were positively correlated with climatic conditions at the beginning of the vegetation period (March to September). Dh was strongly positively correlated (*r* = 0.52) with SMI_1.8_ in May, while Ks was positively correlated (*r* = 0.48) with SPEI-6 of May. In addition, both Dh and Ks were negatively correlated with average spring temperature (*r* = − 0.32 and *r* = − 0.45, respectively) (Supplementary Fig. S7).

For all further analyses, we focused only on six (RW, EW, LW, TVA, Dh and Ks) of the nine initial tree-ring and vessel related variables due to the strong correlations among them (Supplementary Fig. S5) and the common climatic signal present in several pairs of variables. Additionally, we excluded the variables expressed as percentages of the total ring area because their climatic signal was, although strong, not independent and confounded with that of ring-width (Supplementary Fig. S5).

#### Growth and hydraulic function before, during and after drought

**Figure 3:**
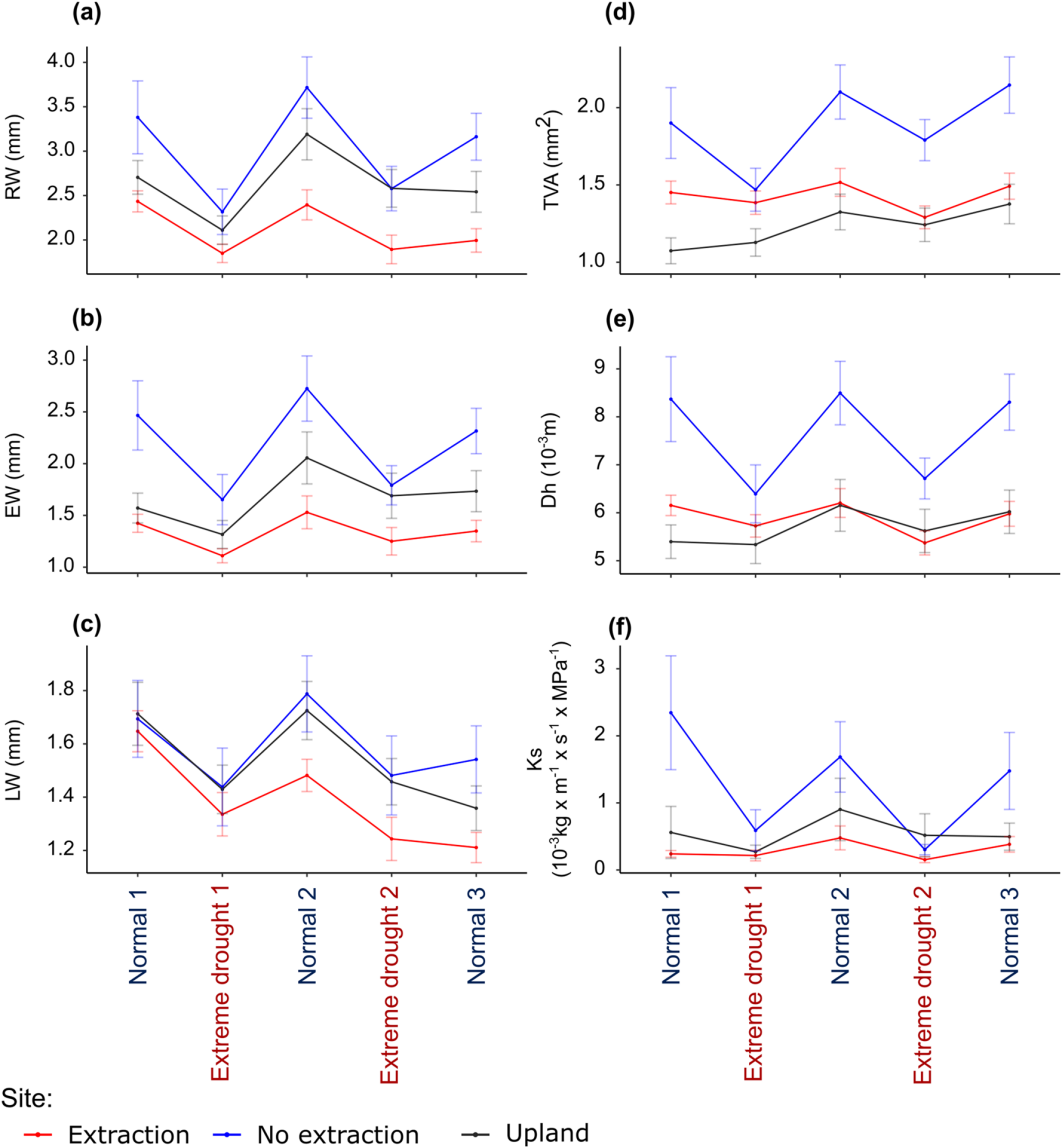
Development of growth: (**a**) ring-width (RW); (**b**) earlywood-width (EW); (**c**) latewood-width (LW), vessel and hydraulic variables: (**d**) total vessel area (TVA); (**e**) hydraulic diameter (Dh); (**f**) theoretical hydraulic conductivity (Ks) in the periods during the two droughts as well as pre, and post-drought (normal). See also Supplementary Fig. S4 for the exact non-dry years considered in the pre- and post-drought periods. Points indicate average values of each ring-width or vessel related variable and bars show the standard error of the mean.

Trees from all sites showed considerable drought-related reductions in all six investigated variables (Fig. 3a–f). RW and EW of oaks at sites with access to groundwater were higher than those of trees at extraction and upland sites. For LW, however, differences among sites were observed mainly in the normal periods following the first extreme drought in 1993. In these periods LW in trees at sites without extraction was wider than in trees at extraction sites. Notably, LW of trees at upland and extraction sites decreased in the normal period following the second extreme drought event (normal 3 in Fig. 3c).

Trees at upland sites had a lower TVA than trees at lowland (extraction or no extraction) sites (Fig. 3d). Still, except for the first drought, trees at sites without groundwater extraction had greater TVA than trees at extraction sites. Finally, extraction and no extraction sites differed clearly regarding Dh and Ks (Fig. 3e,f). Especially in the normal periods (non-dry years), Dh and Ks in trees at no extraction sites were higher than in trees at extraction and upland sites.

#### Growth and hydraulic function before, during and after drought for healthy versus declining trees

**Figure 4:**
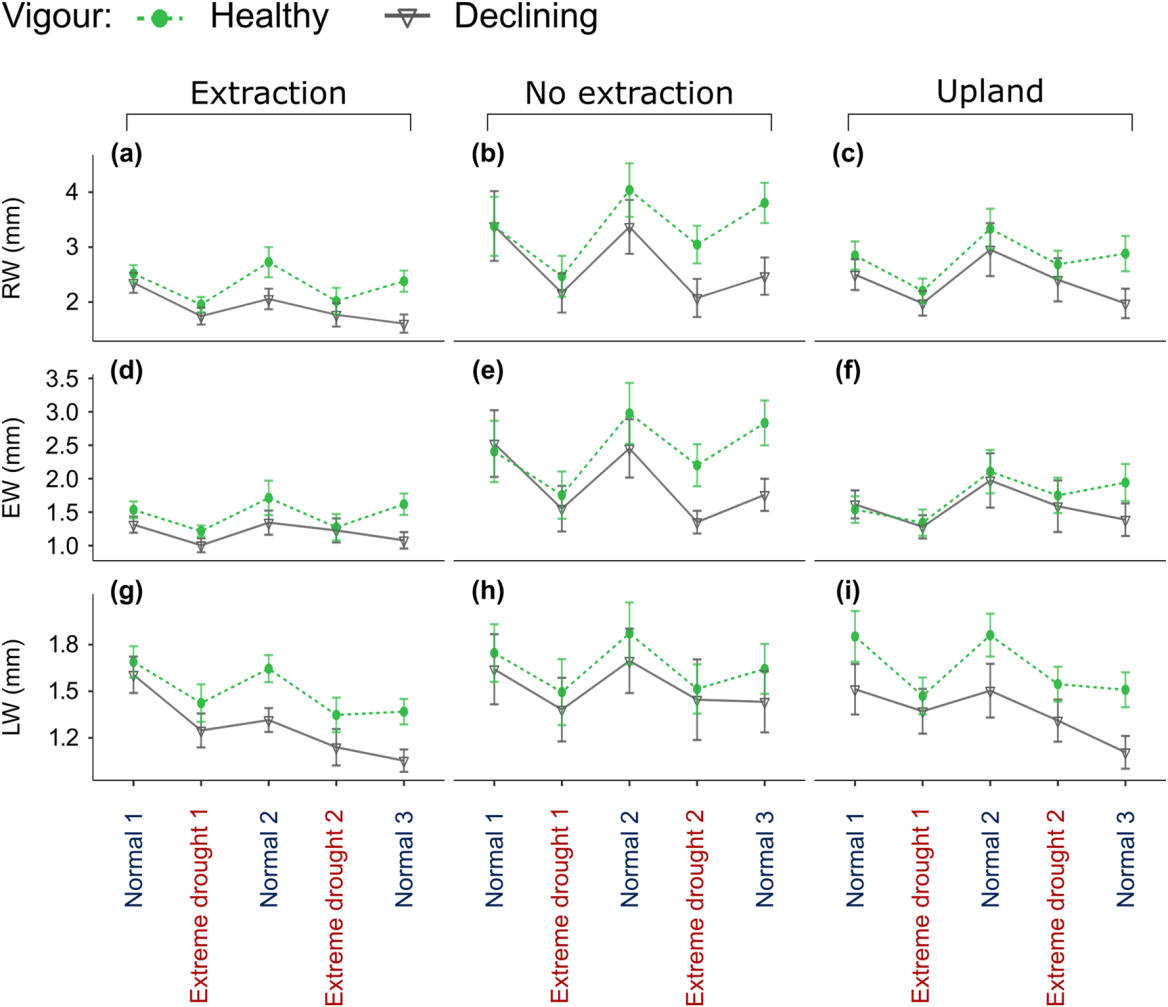
Development of growth variables: (**a**–**c**) ring-width (RW); (**d**–**f**) earlywood-width (EW); (**g**–**i**) latewood-width (LW), in the two droughts as well as pre, and post-drought (normal) periods for trees from different vigour groups and for sites with different groundwater regimes. See also Supplementary Fig. S3 for the exact non-dry years considered in the pre- and post-drought periods. Points show average values of each ring-width variable and bars show the standard error of the mean.

There were marked differences between healthy and declining trees concerning all three growth variables at all site-types (Fig. 4a–i). Not surprisingly, growth was higher in healthy than in declining trees. At extraction sites, these differences increased following the first extreme drought in 1993. Also, for the trees at the extraction sites we observed marked differences in LW which increased over time (Fig. 4g). At both extraction and upland sites, there was a steep decrease for declining trees in all ring-width variables and especially in LW following the second extreme drought.

**Figure 5:**
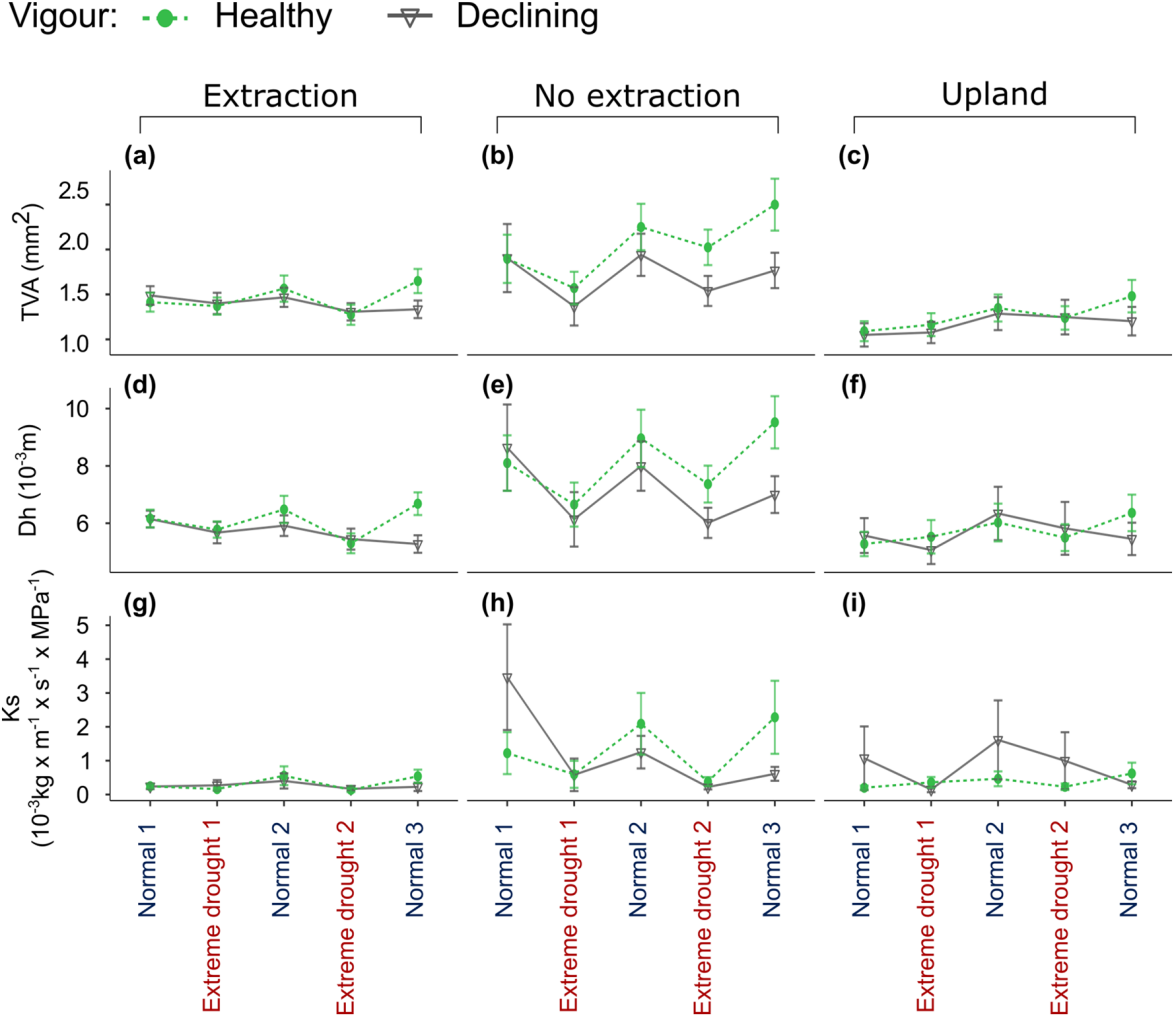
Development of vessel-related variables: (**a**–**c**) total vessel area (TVA); (**d**–**f**) hydraulic diameter (Dh); (**g**–**i**) theoretical hydraulic conductivity (Ks), in in the two droughts as well as pre, and post-drought (normal) periods for trees from different vigour groups and sites with different groundwater regimes. See also Supplementary Fig. S3 for the exact non-dry years considered in the pre- and post-drought periods. Points show average values of each vessel related variables and bars show the standard error of the mean.

Overall, there were clear differences between healthy and declining trees at no extraction sites in all three vessel related variables (Fig. 5b,e,h). These differences tended to increase after the first drought. Healthy trees at extraction sites demonstrated greater values than declining ones for all three vessel-related variables but only following the second extreme drought (Fig. 5a,d,g). Similarly, declining and healthy trees at upland sites did not show noticeable differences regarding TVA and Dh except in the third normal period (Fig. 5c,f). Surprisingly, declining trees at no extraction and upland sites showed greater Ks prior to the first drought compared to healthy trees (Fig. 5h,i).

#### Responses of tree growth and anatomy to drought

**Figure 6:**
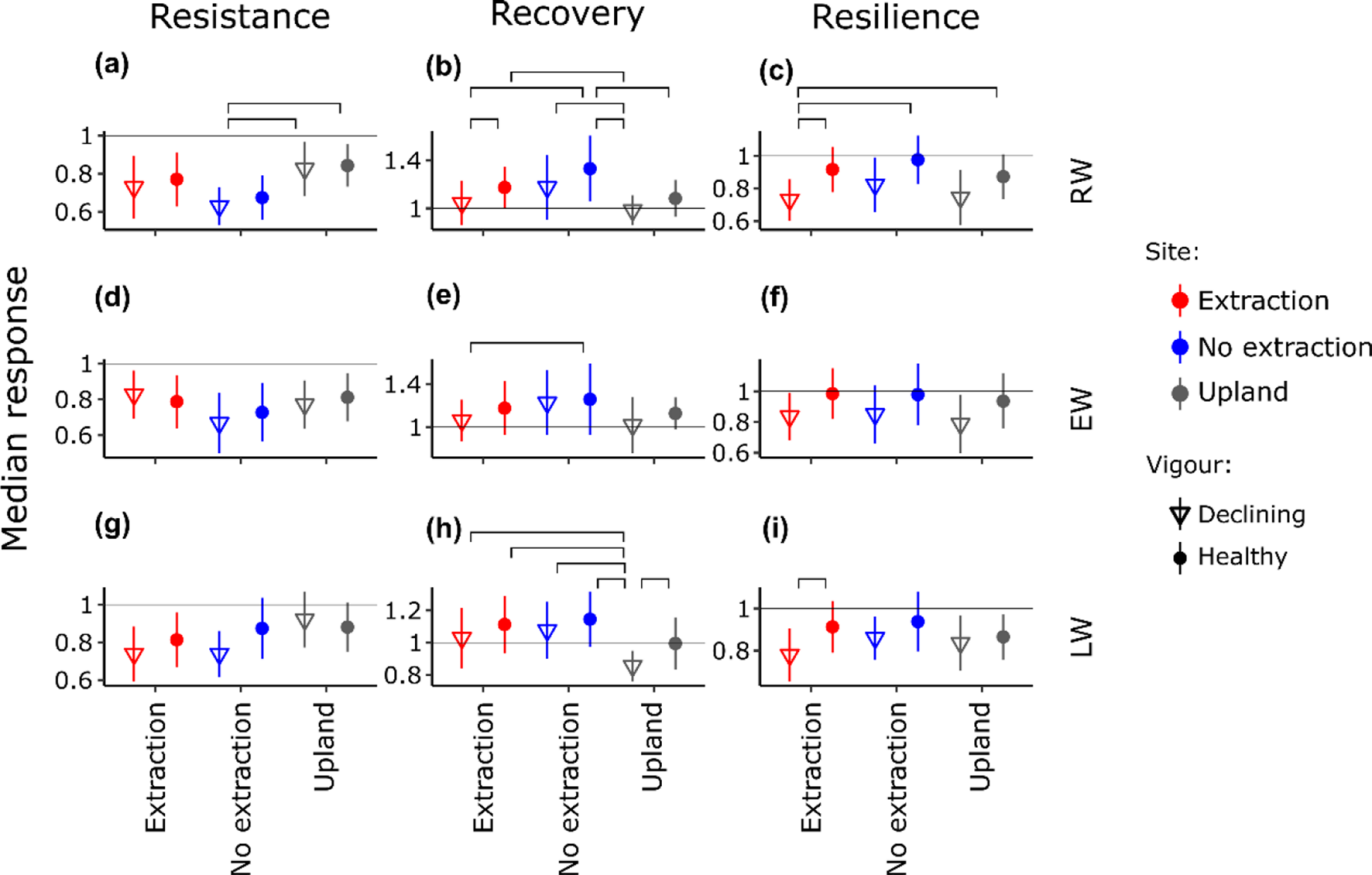
Median components of resilience (resistance: (**a**), (**d**) and (**g**); recovery: (**b**), (**e**) and (**h**); and resilience: (**c**), (**f**) and (**i**) for ring width variables (ring-width (RW); earlywood width (EW); latewood width (LW)) of trees at different sites (red for extraction, blue for no extraction and grey for upland sites). Points denote healthy trees and triangles declining trees. Bars denote median absolute deviation. Brackets connect statistically significant different groups (*p* < 0.05).

**Figure 7:**
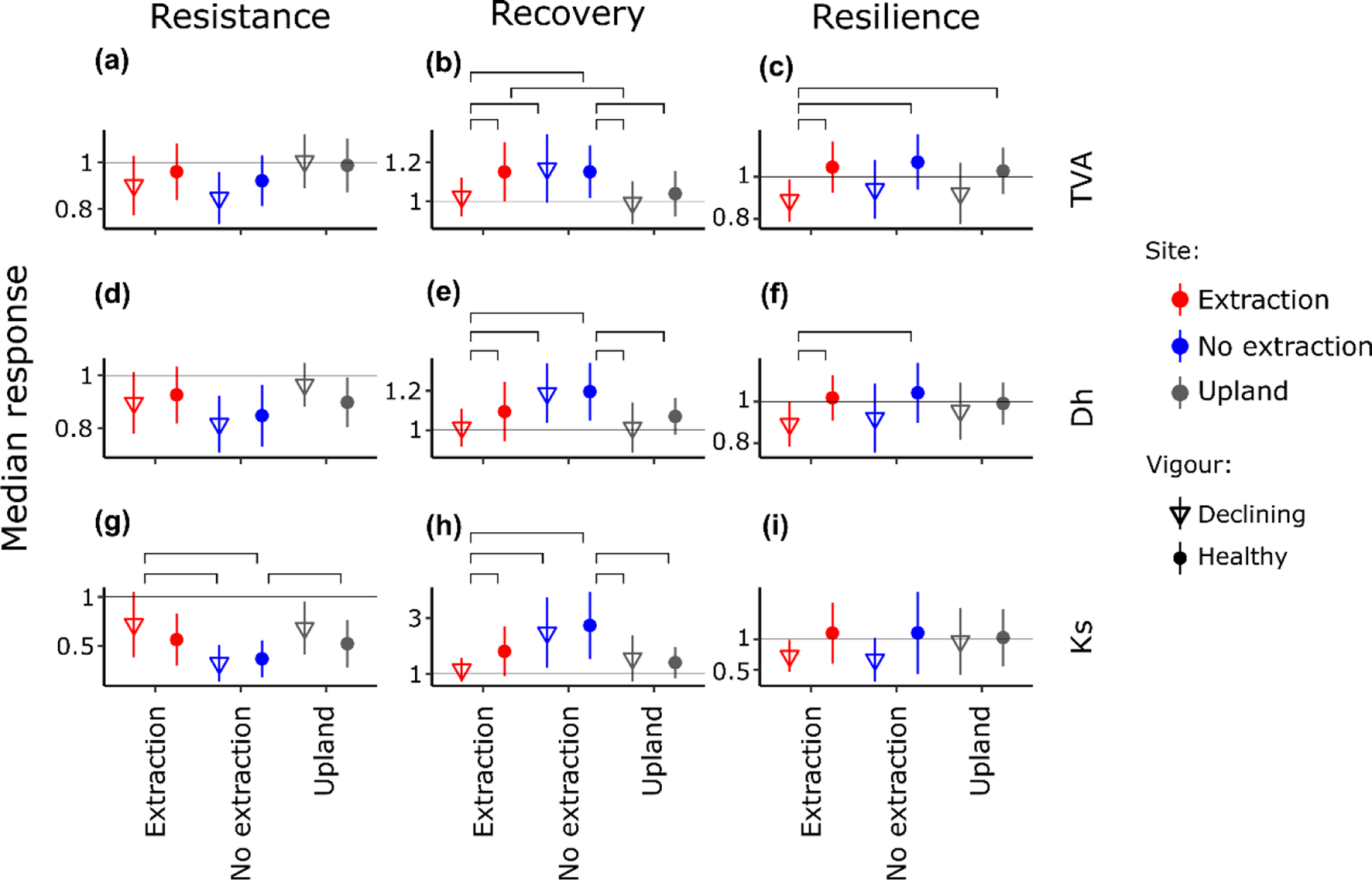
Median components of resilience (resistance: (**a**), (**d**) and (**g**); recovery: (**b**), (**e**) and (**h**); and resilience: (**c**), (**f**) and (**i**)) for vessel related variables (total vessel area (TVA); hydraulic diameter (Dh); and hydraulic conductivity (Ks)) of trees at different sites (red for extraction, blue for no extraction and grey for upland sites). Points denote healthy trees and triangles declining trees. Bars denote median absolute deviation. Brackets connect statistically significant different groups (*p* < 0.05).

Resilience components were significantly different among sites and tree vigour groups for all 6 ring-width and vessel related variables (Figs. 6, 7). These differences were more pronounced for the indices of recovery and resilience and weaker for resistance. Trees at no extraction sites showed the lowest resistance to drought regarding almost all variables. However, differences among sites and tree vigour groups in resistance were only significant for RW and Ks. Healthy trees at no extraction sites had significantly lower RW resistance than both vigour groups at upland sites (Fig. 6a). Remarkably, resistance regarding Ks was higher (i.e. less pronounced reductions in Ks during drought compared to the pre-drought levels) in declining trees from extraction sites and trees from upland sites regardless of vigour than in trees at no extraction sites (Fig. 7g).

Post-drought recovery of ring width variables was highest in healthy trees from no extraction sites (Fig. 6). Recovery of vessel related variables was highest in both healthy and declining trees at no extraction sites (Fig. 7).

The differences among sites regarding resilience were less pronounced than the differences in recovery for all 6 ring-width and vessel related variables. However, differences in resilience were more noticeable between tree vigour groups, with healthy trees demonstrating higher resilience than declining trees at all sites and for almost all variables. Declining trees at the extraction sites showed the lowest resilience in RW (Fig. 6c) and TVA (Fig. 7c). Resilience of LW was significantly higher in healthy than declining trees at extraction sites (Fig. 6i). Resilience in Dh of trees at extraction sites was significantly lower than the resilience of healthy trees from both extraction and no extraction sites (Fig. 7f). Although there were no statistically significant differences among sites or tree vigour groups, healthy trees featured higher resilience in Ks than declining trees, meaning that healthy trees had a higher ability to restore Ks to pre-drought levels following the drought events (Fig. 7i).

## Discussion

Previous studies have reported strong relationships between growth of pedunculate and sessile oak and different drought indices^20,43–46^. The nine growth and vessel related variables analysed in this study encoded strong and, in most cases, distinct hydro-climatic signals. The two soil moisture anomaly indices from the German Drought Monitor modelling product, which, to our knowledge, were used for the first time as predictors of tree growth and xylem anatomical variability, provided the strongest correlations with most of the analysed growth and vessel related variables. This is not surprising given the importance of soil water availability for plants^47^, yet it has been challenging to assess these relationships owing to the lack of long-term and spatially detailed soil moisture data series. The strong positive correlations observed between RW and average SMI for the period from March to August or individual months of the vegetation period indicate that radial growth of oaks was strongly controlled by soil water availability during the whole vegetation period.

In contrast to RW, EW and LW showed a more immediate climatic signal. EW was strongly correlated with climatic conditions in the early vegetation period as demonstrated by the strong positive correlations with SMI_1.8_ in June, SPEI-6 of June, and average spring precipitation (March to May). Variability in LW was more strongly related to soil moisture anomalies later in the summer as demonstrated by the strong positive correlations with SMI_1.8_ in July and the average of SMI_1.8_ of summer months. Additionally, average precipitation during spring and summer months was strongly positively related to LW.

Variables related to the size of earlywood vessels and the two indicators of hydraulic conductivity were strongly related to climatic conditions during short (one to three months-long) time windows. In accordance with other studies^39^, we found a strong negative correlation between MVA and SMI_1.8_ of June. This suggests that MVA actually decreases in years with favourable late spring and early summer conditions. It has been reported that vessel enlargement in pedunculate oaks from the north-western Iberian Peninsula continued until early July^41^ and beginning of May in central Netherlands^48^. Under these conditions, trees might continue to produce earlywood vessels in early summer, which are however of smaller size, thus reducing the mean size of earlywood vessels. The positive relationships observed here between SMI_1.8_ in May and TVA, Ks and Dh corroborate findings of previous studies that reported a direct dependence of earlywood vessel size (and as a result of water conductivity) on water availability during the time of their formation^39,40,49^.

Correlations between radial growth or vessel related variables with climatic conditions in the year prior to ring formation were significant but weaker than those observed in relation to climatic conditions of the current year. The strong correlations between RW, EW and vessel related variables with average precipitation in the summer or vegetation period of the previous year have been attributed to the high dependence, especially of ring-porous species, on stored carbohydrate reserves to resume growth in spring^39,40,50^. Additionally, latewood vessels formed in the previous year might be hydraulicly active and play a crucial role for early spring growth before the newly formed vessels are functional^51^. However, another possible explanation might be that high soil moisture in late summer and autumn could be also carried over winter to the following spring affecting cambial activity and carbon uptake early in the growing season (e.g.^52^). Radial growth in ring-porous species begins already before budburst with large parts of the earlywood being formed with carbohydrate reserves accumulated in the previous year^32,33,48^. Therefore, anatomical variables related to the size of earlywood vessels might be more indicative of climatic conditions in spring than EW width.

Our results suggest that the combination of radial growth and wood anatomical variables can provide complementary information that improves our understanding of tree responses to climatic conditions at different times of the vegetation period or even in the year before.

Trees at no extraction sites had overall higher growth, produced vessels of greater size and exhibited higher hydraulic conductivity than trees from upland and groundwater extraction sites. Previous studies have yielded similar results suggesting increasing above-ground tree productivity, xylem hydraulic conductivity and water use efficiency with decreasing distance to the groundwater table^20,53,54^. Further, an overall higher variability in growth and anatomical variables in years of differing climatic conditions; i.e. reduced growth in drought years and increased growth in non-dry periods, was observed for trees at no extraction sites compared to trees at the extraction and upland sites (Fig. 3). This high variability combined with overall higher growth and hydraulic performance in trees at sites without extraction suggests that access to groundwater enhances plasticity in response to changes in water availability^53–55^. Importantly, plasticity in response to fluctuations in water availability is of high relevance for predictions of tree responses to projected future drought frequency and severity^27,56,57^. This high plasticity should not be misinterpreted as low “stability” of radial growth^20^.

Differences among trees from the three site types, as indicated by all tree-related variables, were most pronounced in the non-dry periods (pre- and post-drought periods in Fig. 3). In years with favourable climatic conditions, larger vessels lead to more efficient water transport and hence higher photosynthetic performance of trees^30^. At the same time, large vessels pose a higher risk of cavitation and thus make trees more vulnerable to soil water deficits^58–60,89,90^. Hence, even trees at sites with adequate access to water are highly sensitive to drought stress in periods with below average precipitation and groundwater levels. This could be the case if, for example, springs with increased water availability (when large earlywood vessels are formed), are followed by extreme summer drought (increased risk of summer cavitation).

When analysing trees from both vitality groups jointly, the differences among sites observed in resistance and recovery from drought were similar across all growth and anatomical variables. In accordance with^6^, our results demonstrate that access to groundwater facilitated recovery of growth and hydraulic functioning of trees in years following droughts. Surprisingly, trees at extraction and upland sites demonstrated higher resistance regarding most variables than trees at no extraction sites (see also Supplementary Fig. S9). However, trees at sites without water extraction had greater absolute growth levels (RW, EW and LW) and TVA, Dh and Ks than trees at the extraction and upland sites in normal periods and to a lesser extent during drought. Therefore, the lower resistance observed in trees with access to groundwater may be explained by the greater growth and hydraulic performance in normal periods, which can then experience greater reductions during drought.

Our results suggest that trees with access to groundwater are more responsive to both periods of favourable and unfavourable conditions than oak trees without groundwater access. Ample availability of water during favourable conditions leads to increased productivity that helps to compensate drought losses (high recovery)^20,61,62^. The resulting higher variability of growth-related variables should therefore not be regarded as an indication for higher susceptibility to drought.

Our results point to some possible explanations for the greater proportion of declining and dying trees at sites with groundwater extraction. Different and potentially interacting mechanisms might have led to loss in tree vitality here. Three mechanisms, which are related to carbon depletion, hydraulic failure and reduced defence capacity against biotic attacks, have been recently proposed to act interdependently in the process of drought-related tree-decline and mortality^63,64^. In our study, declining trees from all sites showed a long-term deterioration of growth and lower hydraulic capacity especially in the post-drought periods. These differences in growth and theoretical hydraulic conductivity between healthy and declining trees were clearly reflected in the three components of resilience (resistance, recovery and resilience) used to quantify tree responses to extreme drought events. Resilience of TVA, Dh and Ks was incomplete in declining trees whereas it was complete in healthy trees. It appears that declining trees were no-longer capable to rebuild their hydraulic system following extreme drought events. Extreme drought events in the past have been found to cause long-lasting effects on the hydraulic system and might have influenced the responses of trees to the most recent drought events^65^.

Long-term deterioration of growth in declining trees at extraction sites was most pronounced in latewood width. This indicates low water availability and concomitantly reduced CO_2_ fixation in trees at the time of latewood formation. In addition, the lower resilience of several anatomical variables (TVA, Dh and Ks) to drought suggest that tree vitality decline at water extraction sites was related to a long-term reduction in C uptake and storage, which results in the inability to fully restore hydraulic function after drought. Under normal or favourable growing conditions, ring-porous oaks use carbohydrate reserves from previous years to restore hydraulic function^32,33^. However, older reserves may be used by trees during and following harsh growing conditions such as multi-year drought events^66^. Here, trees at groundwater extraction sites had a reduced ability to increase radial growth (RW and LW) in periods of favourable growing conditions. This may indicate that trees are also not able to restore reserves of non-structural C. As demonstrated by the overall low total vessel area (TVA) and potential hydraulic conductivity (Ks) at extraction sites, reduced water availability following the onset of groundwater extraction might have forced trees to produce vessels of smaller size. A reduction of vessel size might safeguard trees against cavitation during soil water deficits but carries the risk of reduced assimilation. An unfavourable C balance may also result in weakened defence mechanisms of trees to biotic attacks^63,64^. However, we were not able to test these interactions and their effects on growth and hydraulic function.

While long-term water deficits may explain the mechanisms behind the observed reductions in tree vitality at extraction sites, different mechanisms might be responsible for the decline in tree vigour at no extraction and upland sites. Remarkably, declining trees at sites that were not influenced by groundwater extraction demonstrated higher theoretical Ks prior to the first extreme drought in 1993. The capacity of xylem conduits to transport water is associated with vessel size^60^. While large conduit dimensions might be advantageous in years with adequate water availability^58^, they are prone to embolism during extreme soil water deficits^59,67,68^. Declining trees from no extraction and upland sites might have thus experienced more frequent hydraulic failure in the first extreme drought of 1993^58^. Such an impairment of the hydraulic system might lead to slow or incomplete tree recovery from drought^58,69^, which was also observed at these sites for growth and hydraulic function of declining trees.

## Conclusions

Our results suggest that the combination of chronic (GW extraction) and abrupt water deficits (extreme droughts) resulted in tree vitality losses and probably also a higher risk of tree mortality. Other studies have shown that tree mortality can eventually occur after long periods of reduced or declining growth, triggered by single or recurring droughts^65,70^ as indicated here by the reduced ability to restore growth and hydraulic capacity after extreme droughts in declining trees. Access to groundwater was advantageous for growth and hydraulic capacity under both favourable and unfavourable climatic conditions. Trees with access to groundwater showed high plasticity in growth and hydraulic capacity during droughts and more rapid and complete recovery after drought. This might be due to both higher availability of water in the post-drought period and higher carbon reserves stored in pre-drought periods. However, under extreme droughts even trees at sites without groundwater extraction might suffer losses in growth and hydraulic capacity. Our study demonstrates that the vitality of groundwater-fed forest ecosystems may be severely impaired through the combination of groundwater extraction and increasing drought frequency and intensity.

## Material and methods

### Study sites and sampling

For the purposes of this study, we utilized tree cores of *Q. robur* and *Q. petraea* that were analysed to assess climate-growth relationships by Skiadaresis et al.^20^. The material was collected from oak dominated groundwater-fed forest stands in three regions (close to the city of Freiburg, Emmendingen and Lampertheim) in the Upper Rhine Valley in south-western Germany (Supplementary Fig. S1).

In each region, oak stands were selected from sites with three different groundwater regimes comprising (a) stands close to wells where groundwater has been extracted over several decades (extraction sites), (b) nearby stands without groundwater extraction (no extraction sites), and (c) sites on free draining soils with trees not influenced by groundwater (upland sites). Upland sites were dominated by *Q. petraea* while extraction and reference sites were dominated by *Q. robur*. In addition to the stands sampled by Skiadaresis et al.^20^, we included nine younger stands (one stand per region and site type) that were established after the onset of groundwater extraction to include trees from all developmental stages (Supplementary Fig. S3). The average annual depth of the groundwater table during the period following the onset of groundwater extraction at extraction sites ranged between 5 and 25 m below surface, while at no extraction sites average annual depth of the groundwater table ranged between 1 and 2 m below surface (see also Table 1 in Skiadaresis et al.^20^). To test whether trees from different sites had used different sources of water (precipitation vs. deeper groundwater) we analysed the oxygen isotope composition (δ^18^O) in wood samples obtained from a wet and a dry year per tree. The slightly lower δ^18^O observed in tree-rings from no-extraction sites compared to extraction sites during wet years (Supplementary Fig. S2) suggests that trees at no extraction sites had indeed utilized groundwater (low δ^18^O values) as an additional source of water^71^. Our study design resulted in a total of 27 stands (12, 7 and 8 stands at extraction, no extraction and upland sites, respectively, Supplementary Fig. S1). Stand age (in 2016) ranged from 25 to 181 years at extraction sites, from 25 to 160 at no extraction sites and from 24 to 181 at upland sites. In Lampertheim, two extraction sites were selected because of the large extent of the forest area affected by groundwater extraction.

Twenty dominant or co-dominant trees per stand were selected, except in the LC65 stand (see Supplementary Fig. S1 for the meaning of stand acronyms) in Lampertheim, where only nine trees could be sampled resulting in a total of 529 target trees. We measured stem diameter at breast height (DBH at 1.3 m) and height of each target tree. Tree vitality assessment was based on the crown conditions of target trees (see also Supplementary Methods for further details on crown vitality assessment)^72^. Extraction of two increment cores per tree, sample preparation and tree-ring width measurements were performed following standard dendrochronological protocols as described in Skiadaresis et al.^20^. The resulting tree-ring series were visually and statistically crossdated by computing Pearson’s correlations between each tree-ring series and a master chronology developed from all the other series from the same forest stand. Statistical crossdating was performed using the Dendrochronology Program Library (*dplR*)^73,74^ in R^75^.

### Wood anatomy analyses

We selected one of the two cores from eight trees per stand (216 cores in total) for wood anatomical analyses. These comprised four healthy (vigour class 0 or 1) and four declining trees (vigour class 2 or 3) to assess differences in growth and hydraulic properties. To ensure representativeness of samples, we selected trees with strongest correlations with average stand tree-ring chronologies. Clean core surfaces were stained black using a permanent marker and then rubbed with white chalk dust to increase the contrast between vessels and other wood cells as described in García-González. & Fonti^76^. These cores were scanned using an Epson Expression 10000XL flatbed scanner at 4800 dpi resolution. The acquired images were then analysed using WinCell pro Version 2013 (Regent Instruments, Quebec). Following a semi-automatic procedure, we measured the position, diameter and lumen area of 404,613 conduits of pedunculate and sessile oaks. For further analyses we considered 300,921 earlywood vessels with a transversal lumen area equal or larger than 0.001 mm^2^ from a total of 11,314 annual tree rings.

Owing to differences in stand age, the length of analysed periods varied between 14 and 144 years (Supplementary Fig. S3). Especially in the youngest trees, we excluded on average the first 10 years of juvenile growth where conduit size and distribution in growth rings were markedly different.

### Data processing and chronology development

Based on the obtained ring-width and vessel related measurements we calculated annually resolved series for nine ring-width and vessel related variables. These comprised ring-width (RW), earlywood-width (EW), latewood-width (LW), mean vessel area (MVA), total vessel area (TVA), total vessel area expressed as percentage of analysed ring area (TVA%) and vessel density (VD). Additionally, based on the Hagen–Poiseuille law, we calculated the hydraulic diameter (Dh)^77^ (Eq. 1) and theoretical hydraulic conductivity (Ks) (Eq. 2)^78^:

1 $$\mathrm{Dh}=\frac{{\sum }_{n=1}^{N}{d}_{n}^{5}}{{\sum }_{n=1}^{N}{d}_{n}^{4}}$$

2 $$\mathrm{Ks}=\left(\frac{\uppi {\uprho }_{\mathrm{w}}}{128\upeta }\right)*\mathrm{VD}*{\mathrm{Dh}}^{4}$$

where $\mathrm{Dh}$ is the weighted average of hydraulic diameter (in m), $\mathrm{d}$ is the diameter of each vessel *n* (area equal or larger than 0.001 mm^2^), $\mathrm{Ks}$ is the theoretical hydraulic conductivity (or specific conductivity sensu [60]) (in kg m MPa^−1^ s^−1^), ${\uprho }_{\mathrm{w}}$ is the density of water at 20 °C (998.2 kg m^−3^ at 20 °C), $\upeta$ is the viscosity of water at 20 °C (1.002 * 10^–3^ Pa s at 20 °C) and VD is the vessel density. Because vessels are typically not perfectly round, vessel diameter was calculated as the average of minimum and maximum diameters^79^.

To assess the overall hydro-climatic sensitivity of ring-width and vessel variables regardless of site conditions, we first built average chronologies for each of the nine variables. Individual tree series of each variable were detrended using a 32-year spline with 50% frequency cut-off. This flexible spline removes the biological trends present in tree-ring and vessel related series while simultaneously preserving annual to decadal variability (high frequency) in growth and conduit dimensions^80,81^. Smoothing splines have been suggested as appropriate methods especially for conduit dimension chronologies^82^. Finally, the biweight robust mean was calculated to develop one superregional residual chronology for each ring-width and vessel variable. The quality of each detrended chronology was assessed by calculating several descriptive statistics commonly used in dendrochronology (Mglk, EPS, Rbar and SNR) (see also Supplementary Table S2 and Supplementary Methods).

### Climate, soil moisture variables and drought indicators

Monthly resolved temperature and precipitation data were acquired from the German Weather Service (Deutscher Wetterdienst) using the meteorological stations closest to study sites (< 20 km). Based on these meteorological data, we calculated the Standardized Precipitation Evapotranspiration Index (SPEI)^83^ using the *SPEI* package in R^83^ (for further details see also Supplementary Methods).

Additionally, we obtained seasonal soil moisture anomaly information from the German Drought Monitor^84,85^. Monthly series of modelled soil moisture for two soil depths expressed as a soil moisture anomaly index (SMI) (SMI_0.25_ for the upper 25 cm and SMI_1.8_ for the total soil column < 180 cm) were available for the spatial resolution of 4 × 4 km for the period between 1951 and 2016. These SMI were calculated from simulations with the mesoscale Hydrologic Model^86^ driven with observed meteorological data. Monthly series were extracted for the grid points closest to the 27 sampled stands.

### Analysis

#### Hydro-climatic sensitivity of ring-width and vessel chronologies

Hydro-climatic sensitivity of ring-width and wood anatomical variables were assessed by calculating bootstrapped Pearson’s correlation coefficients between the detrended ring-width and vessel chronologies and monthly as well as seasonal means for all hydro-climatic variables, using the package *treeclim* in R^87^. Correlation functions were calculated for separate months from March of the previous year to October of the current year, as well as for seasonal means of the previous and current year (Supplementary Figs. S6–S8). For this analysis we considered the period between the years 1951 and 2016, for which SMI were available.

#### Identification of drought events

Drought events at our study sites were identified by classifying vegetation season SMI_1.8_ averages for the time-period between 1977 (8 years after the onset of groundwater extraction in Lampertheim) and 2015 into ‘extreme’ (lowest 5%), ‘severe’ (5–10%) and ‘moderate’ dry years (11–20%) (see also Supplementary Methods). Based on this definition the year 1993 (extreme drought 1, in Supplementary Fig. S4) was identified as extremely dry in all three regions (Lampertheim, Freiburg & Emmendingen). In the region of Freiburg, the year 2003 was identified as the second most extreme drought event (Extreme drought 2 in Supplementary Fig. S4). In Lampertheim and Emmendingen the second most extreme event was identified one year later, in 2004.

#### Assessing responses to drought

To assess growth and anatomical responses of trees to extreme drought events, we used the components of resilience (resistance, recovery and resilience) as proposed by Lloret et al.^24^. These resilience indices have been widely used to quantify responses of tree growth to drought and recently also for xylem anatomical variables^38^. ‘Resistance’ describes a tree’s capacity to absorb changes in radial growth during a drought event and is calculated as the ratio of growth during drought divided by mean growth in a pre-drought period. The index ‘recovery’ is calculated as the ratio of mean growth in a post-drought period divided by growth in the drought year, while ‘resilience’ represents the ratio of average post-drought tree growth divided by mean growth in the pre-drought period. To overcome limitations of a predefined reference period (pre- and post-drought)^88^ we used a slightly modified approach from the one proposed by Lloret et al.^24^ for calculating pre- and post-drought means (hereafter referred to as normal periods). These were defined as the average raw value of each growth or vessel-related variable in all non-dry years (i.e., excluding values in moderate and severe dry years) occurring before, between and following the two identified extreme droughts. The first normal period was the period between 1977 and 1993, the second normal period was the period between 1994 and 2002 or 2003, while the third normal period was from 2004 (for Freiburg) or 2005 (for Emmendingen and Lampertheim) to 2015 (Supplementary Fig. S4).

We used the Shapiro–Wilk test to test for normality in the distribution of the components of resilience per site and vigour group. As these were not normally distributed, we used the non-parametric Kruskal–Wallis test for statistically significant differences in resilience components (resistance, resilience and recovery) among vigour groups (healthy and declining) and sites (extraction, no extraction, and upland). Trees belonging to the same vigour group and groundwater conditions were pooled across the three study regions. We used Wilcoxon sign tests to perform pairwise comparisons among the three different sites and vigour groups.

## Supplementary Information

Supplementary Information

## Data Availability

The datasets generated and analyzed during the current study are available from the corresponding author on reasonable request.
