# Supplementary material for: Groundwater extraction reduces tree vitality, growth and xylem hydraulic capacity in Quercus robur during and after drought events
Source: Sci Rep. 2021 Mar 4;11:5149. doi: 10.1038/s41598-021-84322-6 (PMC7970862; doi:10.1038/s41598-021-84322-6)
Supplement: Supplementary file 1 — Supplementary Information [file 41598_2021_84322_MOESM1_ESM.pdf]

Supplementary Material

Article title: Groundwater extraction reduces tree vitality, growth and xylem hydraulic capacity in *Quercus robur* during and after drought events

Authors: Georgios Skiadaresis, Julia Schwarz, Kerstin Stahl and Jürgen Bauhus

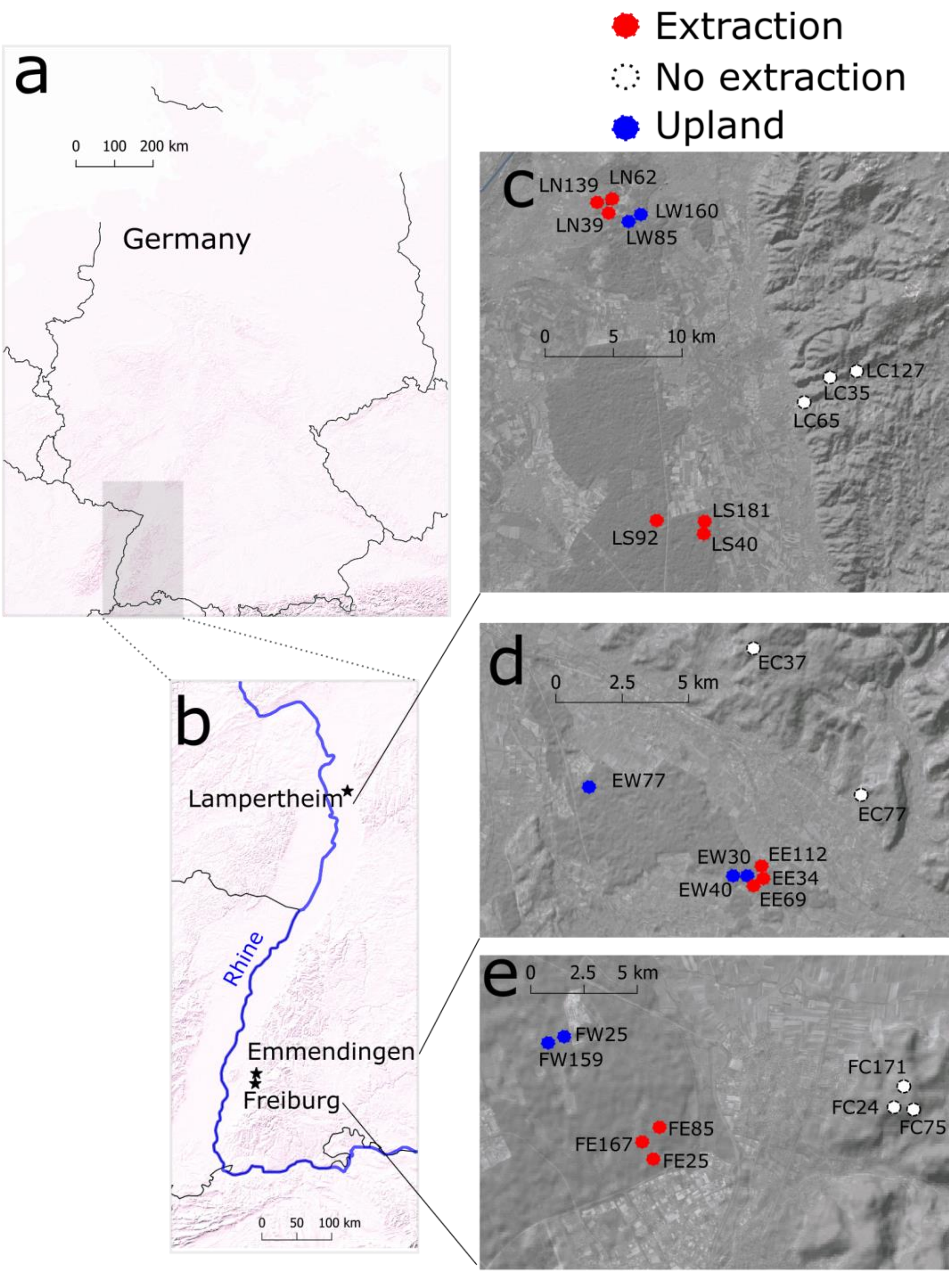

Fig. S1: Location of the study region (a), the three study regions near Freiburg, Emmendingen and Lampertheim (b), and locations of the investigated forest stands in each region (Lampertheim, Emmendingen and Freiburg) (c, d and e). Stand IDs in (c), (d) and (e) are acronyms denoting the study region and site type (first and second letter, respectively) of each investigated stand. F, E, L for Freiburg, Emmendingen and Lampertheim, respectively. W indicates sites without groundwater extraction, C upland sites and E, S and N are extraction sites (E for the extraction sites in Emmendingen and Freiburg, S and N for the Southern and Northern extraction sites, respectively, in Lampertheim). Numbers in Stand IDs indicate the average tree age in each stand. The maps (a-e) were created using QGIS version 3.14-pi (https://qgis.org/en/site/). Satellite images in c-e were obtained using the QuickMapServices Plugin (https://plugins.qgis.org/plugins/quick_map_services/) in QGIS.

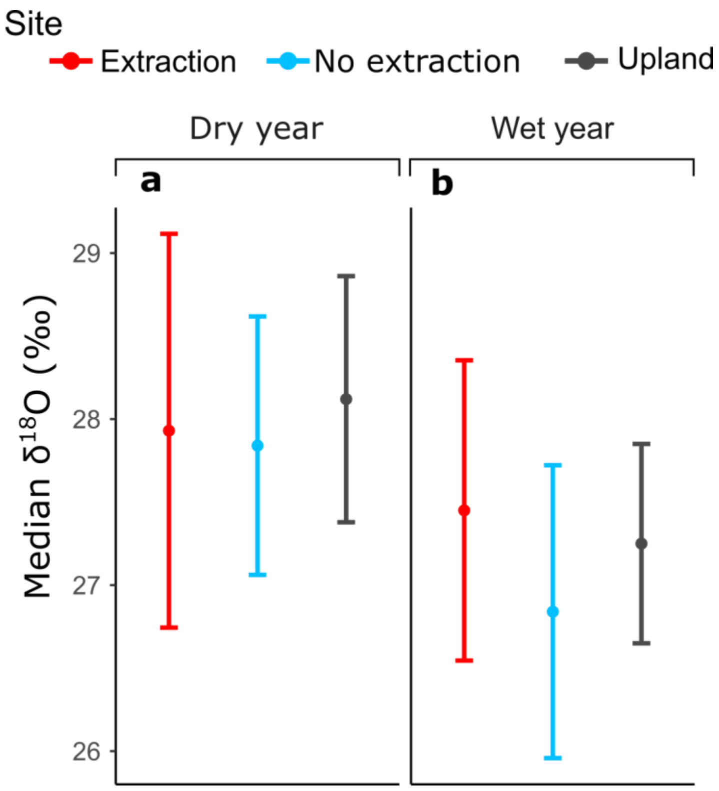

Fig. S2: Median oxygen isotope composition $\delta^{18}O$ (‰) in tree-rings formed in (a): a dry (2003) and (b) a wet-normal year (2016 for Emmendingen and Freiburg, and 2013 for Lampertheim). Bars denote median absolute deviation.

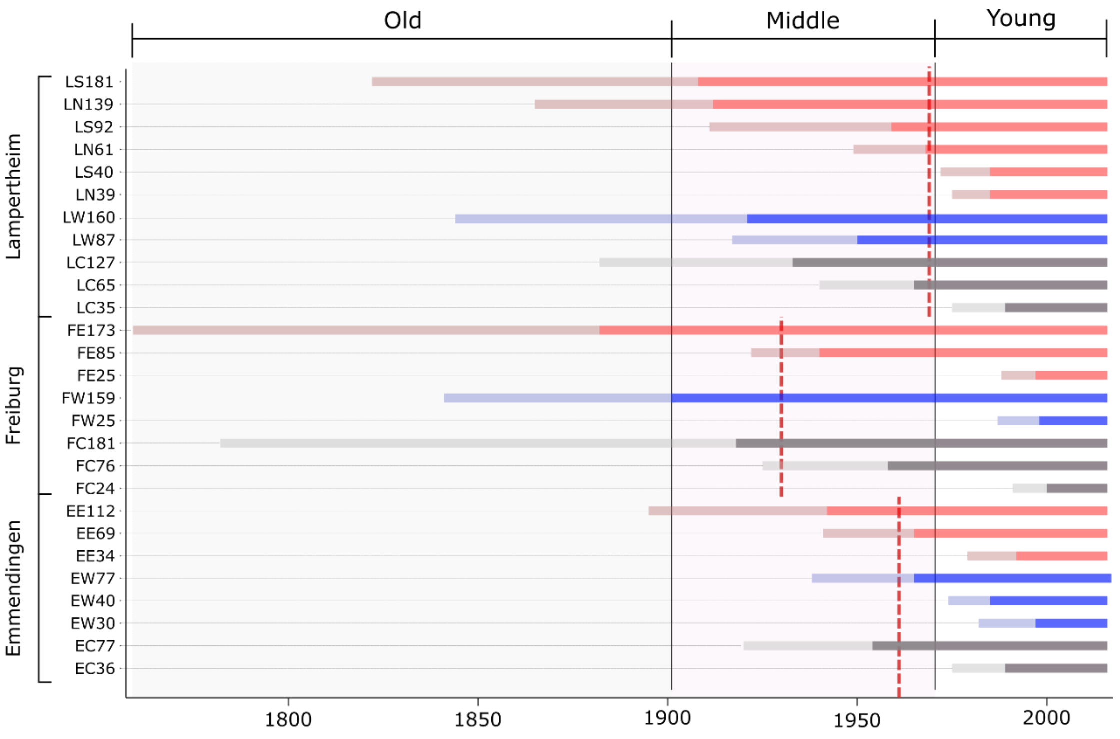

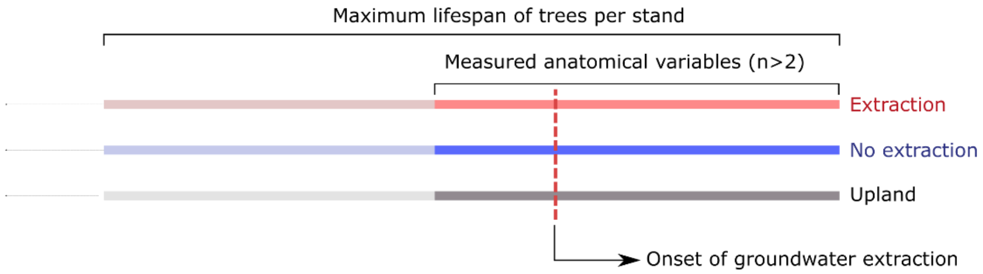

Fig. S3: Livespan of investigated forest stands for the three investigated regions. Colours indicate site types (red for groundwater extraction sites, blue for no-extraction sites and grey for upland sites). Bars with light colours indicate the maximum lifespan of trees per stand while the darker colours indicate the period during which vessel variables where measured. Vertical dashed lines coloured in red indicate the onset of groundwater extraction at the extraction sites. Stand IDs are acronyms denoting the study region and site type (first and second letter, respectively) of each investigated stand. F, E, L for Freiburg, Emmendingen, and Lampertheim, respectively. W indicates sites without groundwater extraction, C upland sites and E, S, and N are extraction sites (E for the extraction sites in Emmendingen and Freiburg, S and N for the Southern and Northern extraction sites, respectively, in Lampertheim). Numbers in Stand IDs indicate the average tree age in each stand.

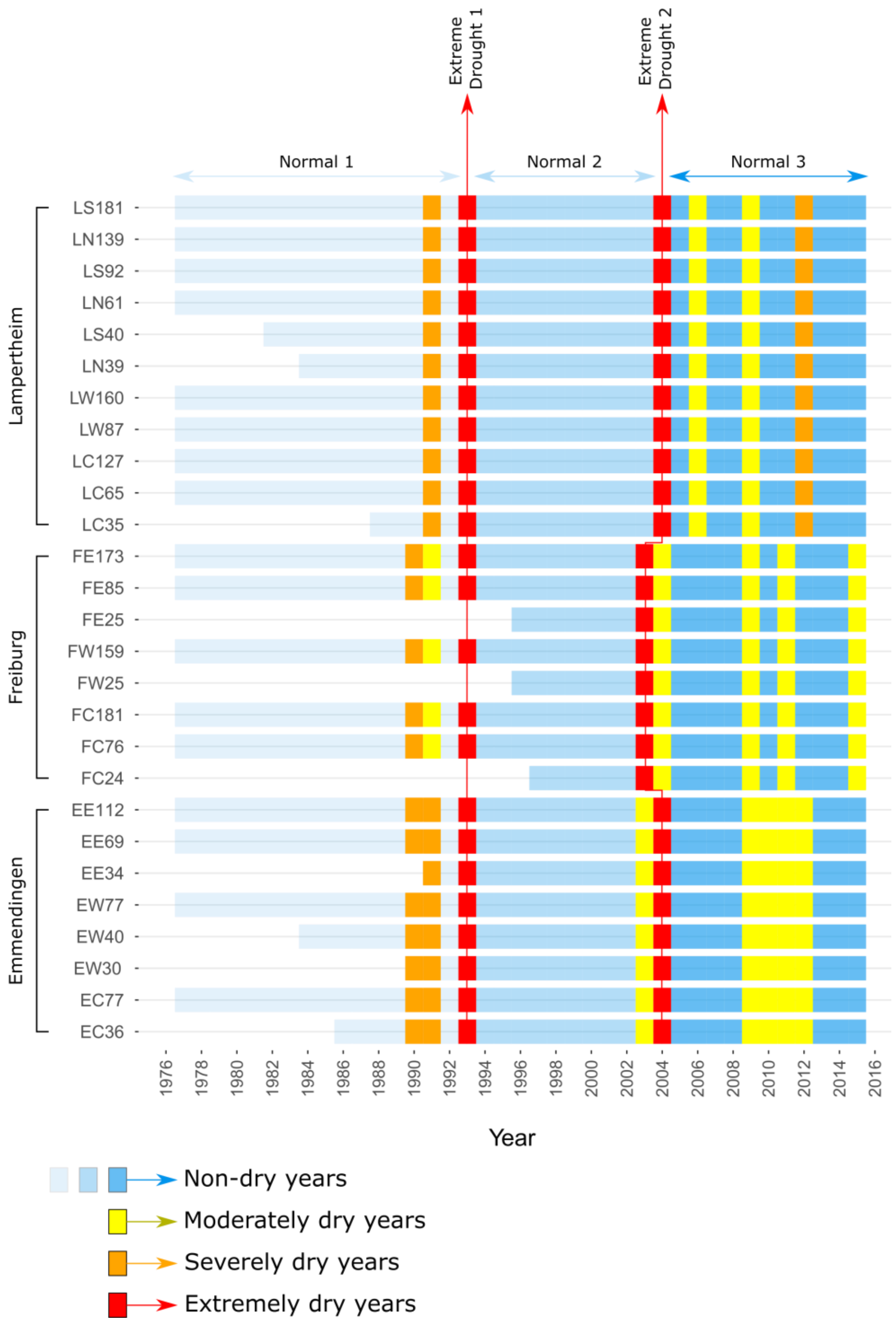

Fig. S4: Identification of drought years based on percentiles of average $SMI_{1.8}$ per stand. Red for extremely dry years (SMI < 5% percentile), orange for severely dry years (5%>SMI>10%) and yellow for moderately dry years (11%>SMI>20%). Blue colours of different intensity indicate non-dry years (SMI>20% percentile) in the three periods: Normal 1 is the period between 1977 and 1993, the period between 1994 and 2002 or 2003 is Normal 2, while the Normal 3 period is from 2004 (for Freiburg) or 2005 (for Emmendingen and Lampertheim) to 2015.

	RW	EW	LW	LW -1 year	Ks	Dh	MVA	TVA	TVA%	VD
RW	1	0.91	0.87	0.29	0.44	0.67	−0.68	0.52	−0.84	−0.67
EW		1	0.62	0.34	0.71	0.86	−0.67	0.73	−0.6	−0.36
LW			1	0.14		0.3	−0.5	0.2	−0.91	−0.91
LW -1 year				1	0.3	0.38	−0.13	0.38	−0.11	
Ks					1	0.92	−0.3	0.88		0.25
Dh						1	−0.38	0.95	−0.19	
MVA							1	−0.09	0.72	0.28
TVA								1	−0.01	
TVA%									1	0.86
VD										1

Fig. S5: Correlations among detrended chronologies of tree ring and wood anatomical variables. Only statistically significant correlations are shown.

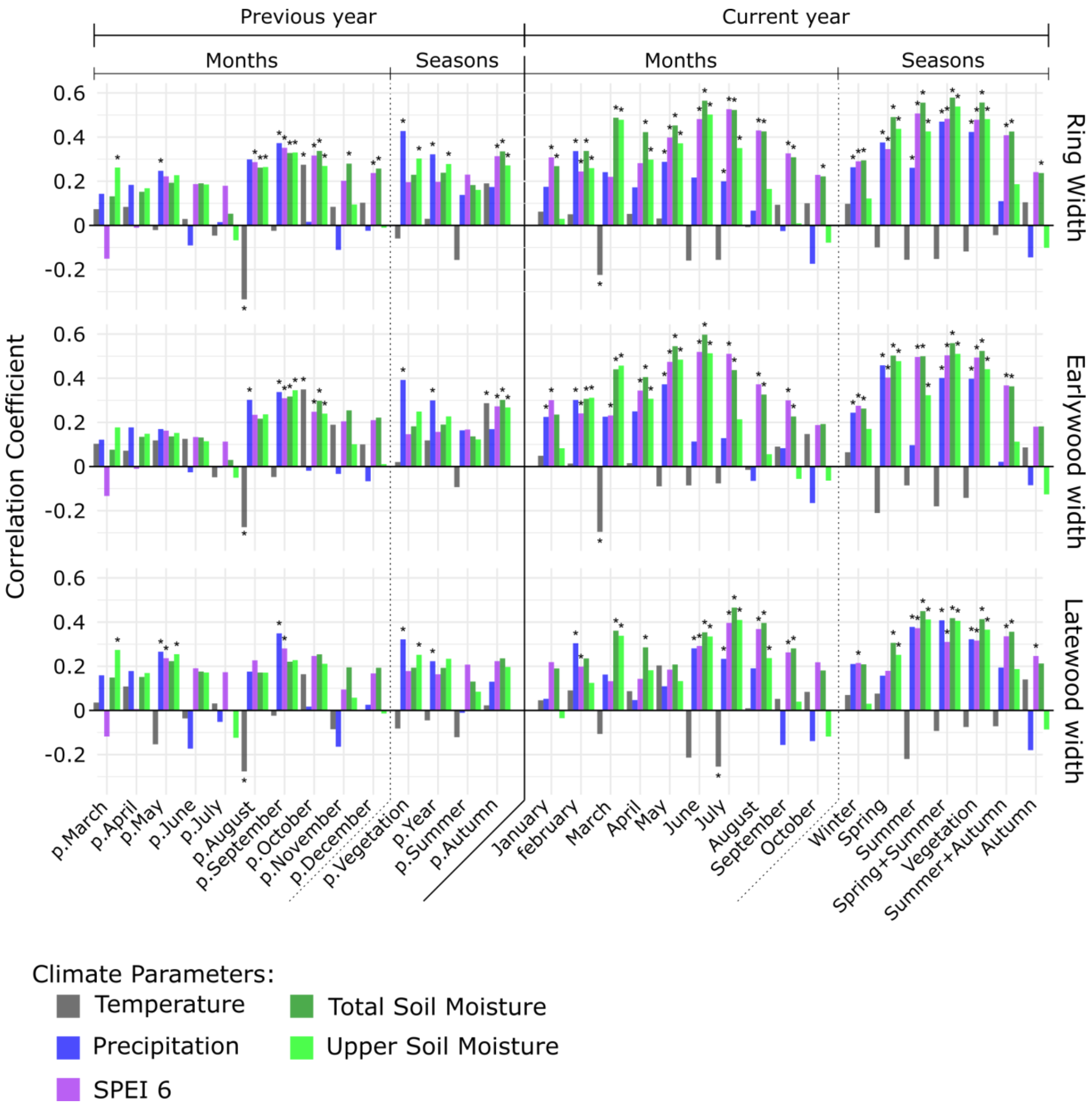

Fig. S6: Correlation coefficients between detrended (spline 32) ring-width chronologies (Total ring width, Earlywood width and Latewood width) and climate variables (Temperature, Precipitation, SPEI 6, total soil moisture index and upper soil moisture index) for the months from March of the previous year to September of the current year and different seasons (Vegetation: from March to September, Year: from January to December, Summer: from June to August, Autumn: from September to November, Winter: from previous year December to current year February). Stars above bars indicate statistically significant correlations. The character p. before months and seasons indicates months and seasons of the previous year. The continuous vertical black line separates correlation coefficients referring to months and seasons of the previous year from those of the current year, while the two dashed lines separate monthly from seasonal correlations.

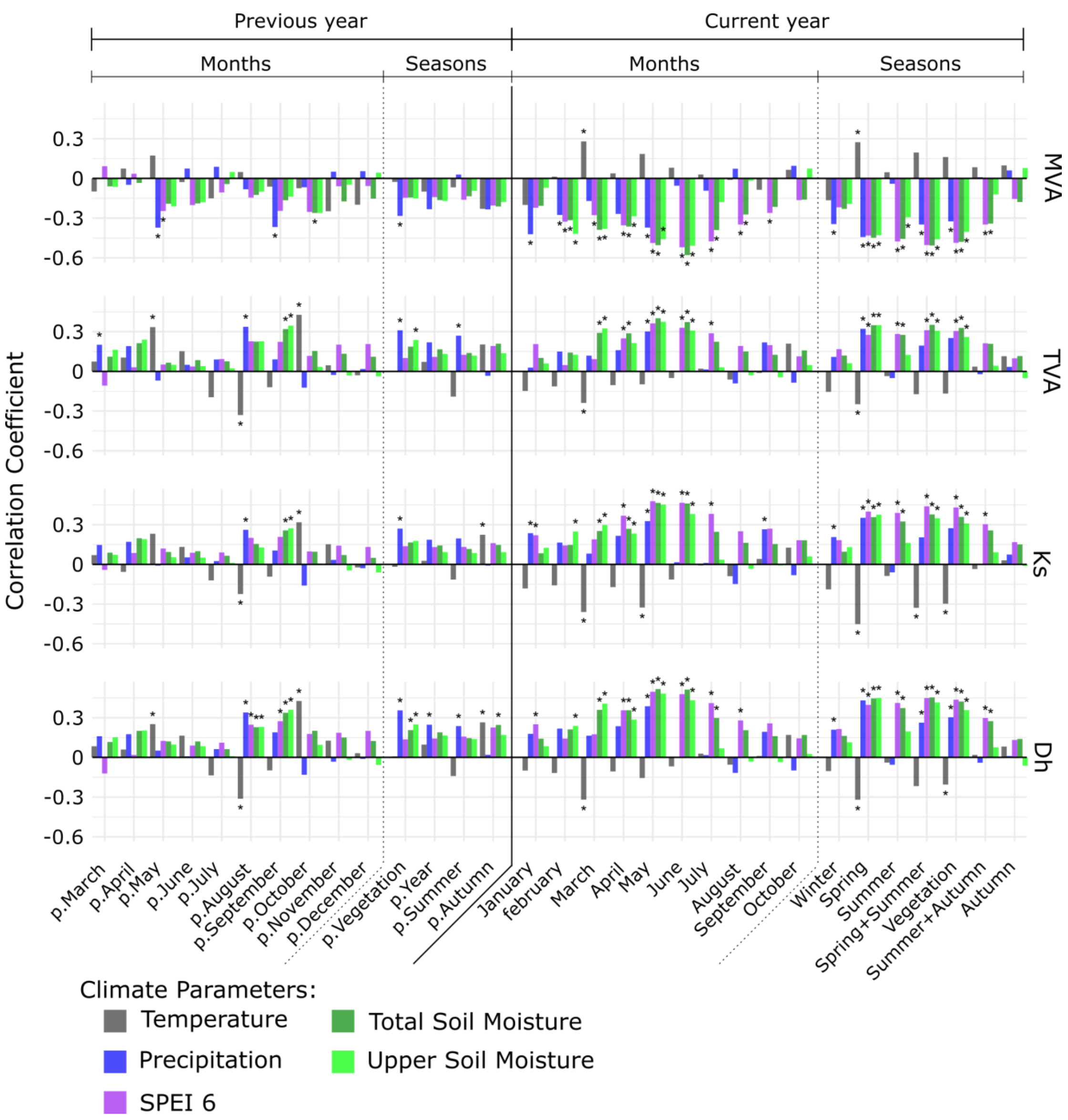

Fig. S7: Correlation coefficients between detrended (spline 32) anatomical variables (MVA: Mean Vessel Area, TVA: Total Vessel Area, Ks: Theoretical hydraulic conductivity and Dh: Hydraulic diameter) and climate variables (Temperature, Precipitation, SPEI 6, total soil moisture index and upper soil moisture index) for the months from March of the previous year to September of the current year and different seasons (Vegetation: from March to September, Year: from January to December, Summer: from June to August, Autumn: from September to November, Winter: from previous year December to current year February). Stars above bars indicate statistically significant correlations. The character p. before months and seasons indicates months and seasons of the previous year. The continuous vertical black line separates correlation coefficients referring to months and seasons of the previous year from those of the current year, while the two dashed lines separate monthly from seasonal correlations.

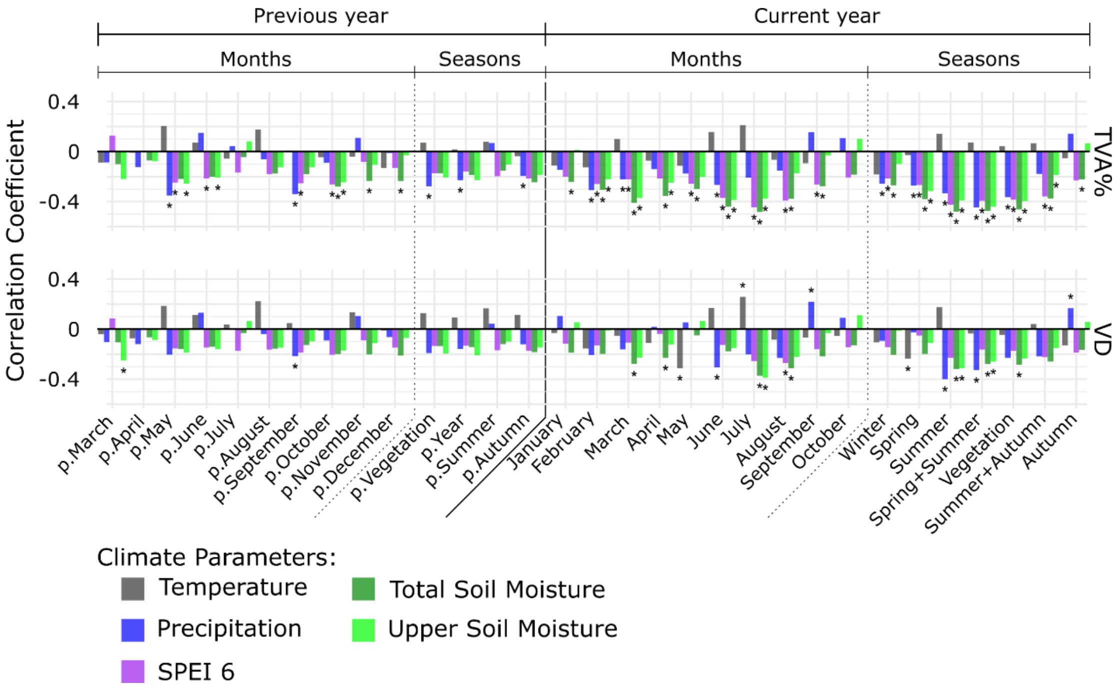

Fig. S8: Correlation coefficients between detrended (spline 32) anatomical variables (TVA%: Total Vessel Area expressed as percentage of ring area and VD: Vessel Density), and climate variables (Temperature, Precipitation, SPEI 6, total soil moisture index and upper soil moisture index) for the months from March of the previous year to September of the current year and different seasons (Vegetation: from March to September, Year: from January to December, Summer: from June to August, Autumn: from September to November, Winter: from previous year December to current year February). Stars above bars indicate statistically significant correlations. The character p. before months and seasons indicates months and seasons of the previous year. The continuous vertical black line separates correlation coefficients referring to months and seasons of the previous year from those of the current year, while the two dashed lines separate monthly from seasonal correlations.

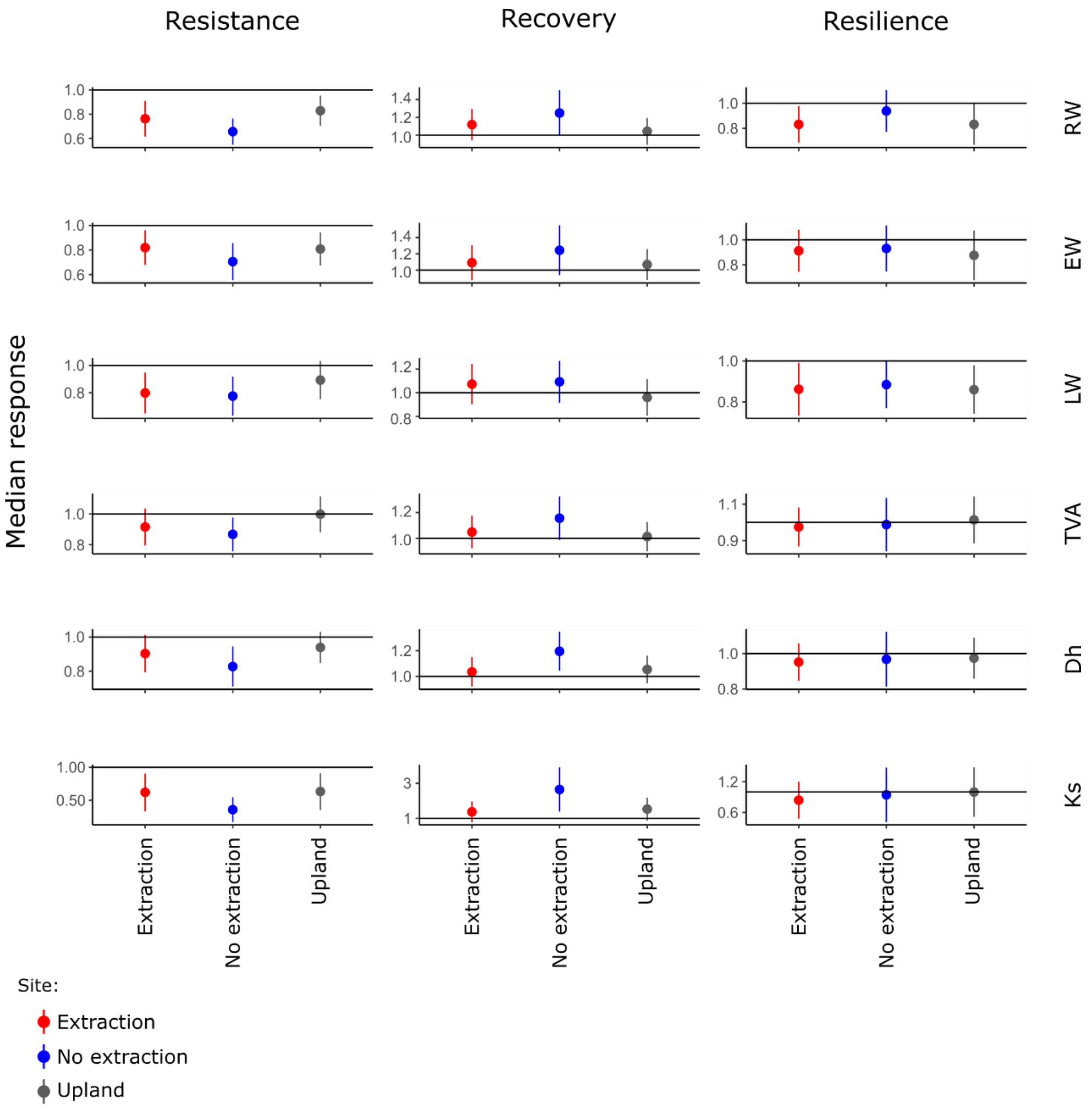

Fig. S9: Median components of resilience (Resistance, Recovery and Resilience) for ring-width (RW), earlywood width (EW), latewood width (LW), total vessel area (TVA), hydraulic diameter (Dh) and theoretical hydraulic conductivity (Ks) of trees at different sites (red for extraction, blue for no extraction and grey for upland sites).

Site	Vigour group	Median height (m)	MAD height	Median Dbh (m)	MAD dbh
Extraction	Declining	**21.5**	8.4	36.1	29.6
Extraction	Healthy	23.2	9.1	32.4	20.1
No extraction	Declining	23.3	7.2	36.7	26.8
No extraction	Healthy	23.8	3.8	38.2	19.7
Upland	Declining	23.7	8.3	35.3	18.4
Upland	Healthy	**29.6**	7.4	43.3	20.4

Table S1: Median and median absolute deviation (MAD) of tree height and diameter at 1.3 m height (dbh) for different sites and vigour classes. Bold numbers indicate the two groups that were significantly different from each other (*p*<0.05, Wilcoxon-test).

		Statistics on detrended chronologies						
Variable	Abbreviation (Units)	EPS	snr	mGLK	Rbar	mean	median	stdev
Ring-width	RW (mm)	0.96	23.7	0.56	0.40	2.86	2.67	1.15
Earlywood-width	EW (mm)	0.93	12.7	0.53	0.28	1.90	1.69	0.91
Latewood-width	LW(mm)	0.88	7.07	0.52	0.23	1.61	1.50	0.63
Hydraulic conductivity	Ks (kg m MPa^{-1} s^{-1})	0.91	10.4	0.54	0.26	0.0007	0.0003	0.0013
Hydraulic diameter	Dh (m)	0.93	13.8	0.55	0.31	0.0068	0.0064	0.0020
Average vessel area	MVA (mm^2)	0.87	6.7	0.52	0.23	0.06	0.06	0.02
Total vessel area	TVA (mm^2)	0.93	12.5	0.55	0.31	1.62	1.57	0.46
Total Vessel Area as percentage of ring area	TVA%	0.95	19.6	0.56	0.36	15.01	14.26	4.92
Vessel Density	VD	0.89	8.2	0.53	0.26	3.43	3.25	1.08

Table S2: Descriptive statistics for the developed chronologies for the period between 1900 and 2017. EPS, expressed population signal; snr, signal to noise ratio; mGLK, mean gleichläufigkeit (synchronicity); Rbar, mean correlation between individual series; stdev, standard deviation.

Supplementary Methods

Crown vitality assessment: Tree vitality was assessed based on the crown conditions of target trees as proposed by Roloff [1]. Vital trees with full crowns and vital fine twigs were assigned to crown class 0; trees assigned to class 1 showed first signs of crown degradation and twig abscission; trees of class 2 were noticeably weakened already; and seriously weakened and dying trees with dead main branches and disintegration of the entire crown were assigned to crown class 3. Selected trees covered all vigour classes present in the studied stands.

Statistics used to assess the quality of developed chronologies: Mean gleichläufigkeit (synchronicity) (Mglk) was used to assess similarity of detrended tree-ring series [2]. The glk() function from the dplR package was used to compute glk (gleichläufigkeit) which performs pairwise comparison of all possible combinations between series. The expressed population signal (EPS) is an indicator of how well a chronology represents a theoretical infinite population [3]. Low values of EPS (commonly <0.85) indicate that the chronologies are dominated by individual tree signals rather than a consistent regional signal [4]. Rbar is the mean correlation between series within a chronology and is a measure of common signal strength of detrended chronologies. The signal to noise ratio (SNR) is a measure of the desired signal in each chronology versus the amount of unwanted information and random variation [4,5].

Climate data and calculation of the Standardized Precipitation Evapotranspiration Index (SPEI): Monthly resolved temperature and precipitation data were acquired from the German Weather Service (Deutscher Wetterdienst) using the meteorological stations closest to study sites (<20 km). For sites near Freiburg and Emmendingen, data (from 1921 to 2016) from one station located within 10 km from the sites were used. For the Lampertheim region, meteorological data were available for the period between 1897 and 2016. Based on these meteorological data, we calculated the Standardized Precipitation Evapotranspiration Index (SPEI) [6] using the SPEI package in R [6]. An accumulation period of 6 months was selected for the calculation of SPEI because we found in an earlier study that this accumulation period correlated best with inter-annual tree-growth variations across the investigated sites [7].

Drought event identification: For the identification of drought events at our study sites we used the SMI1.8 for the time-period between 1977 (8 years after the onset of groundwater extraction in Lampertheim) and 2015. Years were classified based on SMI1.8 averages of the vegetation season into: "extreme drought", "severe drought", "moderate drought" and "not dry" using a percentile approach (see also [8]) to classify years based on their historical frequency. Accordingly, extremely dry years are events with a likelihood of occurrence of < 5% of the time (between 1977 and 2015). Severely dry years have a 5-10% and moderately dry years a 11-20% chance of occurrence. With the percentile approach we identified two extreme drought events for each region (Supplementary Fig. S.4). The year 1993 (extreme drought 1, in Supplementary Fig. S4) was identified as extremely dry in all three regions (Lampertheim, Freiburg & Emmendingen). In the region of Freiburg, the year 2003 was identified as the second most extreme drought event (Extreme drought 2 in Supplementary Fig. S4). In Lampertheim and Emmendingen the second most extreme event was identified one year later, in 2004.

References

1. Roloff, A. Baumkronen: Verständnis und praktische Bedeutung eines komplexen Naturphänomens [Tree crowns: comprehension and practical meaning of a complex phenomenon]. *Ulmer, Stuttgart [original in German]* (2001).
2. Schweingruber, F. H. *Tree rings - basics and applications of dendrochronology* (1988).
3. Wigley, T. M. L., Briffa, K. R. & Jones, P. D. On the average value of correlated time series, with applications in dendroclimatology and hydrometeorology. *J. Climate Appl. Meteor.* **23,** 201–213 (1984).
4. Speer, J. H. *Fundamentals of tree-ring research* (University of Arizona Press, 2010).
5. Cook, E., Briffa, K., Shiyatov, S., Mazepa, V. & Jones, P. D. in *Methods of Dendrochronology,* (ed Cook E. R. & Kairiukstis L. A.) 97–162 (Springer 2013).
6. Vicente-Serrano, S. M., Beguería, S. & López-Moreno, J. I. A multiscalar drought index sensitive to global warming: the standardized precipitation evapotranspiration index. *J. Clim.* **23,** 1696–1718 (2010).
7. Skiadaresis, G., Schwarz, J. A. & Bauhus, J. Groundwater extraction in floodplain forests reduces radial growth and increases summer drought sensitivity of pedunculate oak trees (*Quercus robur* L.). *Front. For. Glob. Change* **2,** 267 (2019).
8. Svoboda, M. *et al.* The drought monitor. *Bull. Am. Meteorol. Soc* **83,** 1181–1190 (2002).
